# Azide Photochemistry in Acrylic Copolymers for Ultraviolet Cross-Linkable Pressure-Sensitive Adhesives: Optimization, Debonding-on-Demand, and Chemical Modification

**DOI:** 10.1021/acsami.2c07385

**Published:** 2022-06-23

**Authors:** Rohani Abu Bakar, Yuman Li, Oliver P. Hewitson, Peter J. Roth, Joseph L. Keddie

**Affiliations:** † Department of Physics, University of Surrey, Guildford, Surrey GU2 7XH, U.K.; ‡ Department of Chemistry, University of Surrey, Guildford, Surrey GU2 7XH, U.K.; § Malaysian Rubber Board, Kuala Lumpur 50450, Malaysia

**Keywords:** pressure-sensitive adhesive, acrylic copolymer, perfluorophenylazides, cross-linking, ultraviolet irradiation

## Abstract

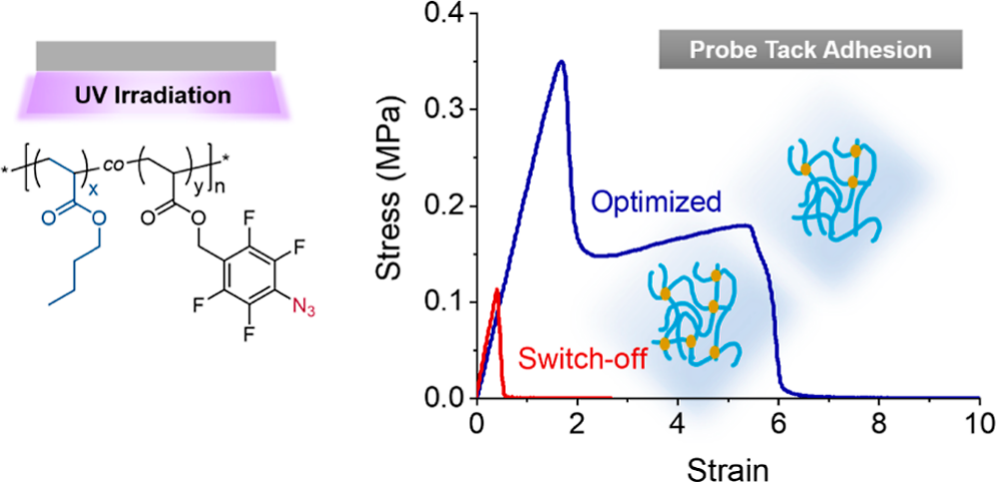

Pressure-sensitive adhesives (PSAs) are usually made from viscoelastic, high-molecular-weight copolymers, which are fine-tuned by adjusting the comonomer ratios, molecular weights, and cross-link densities to optimize the adhesion properties for the desired end-use. To create a lightly cross-linked network, an ultraviolet (UV) photoinitiator can be incorporated. Here, we present the first use of perfluorophenylazide chemistry to control precisely a polyacrylate network for application as a PSA. Upon UV irradiation, the highly reactive nitrene from the azide moiety reacts with nearby molecules through a C–H insertion reaction, resulting in cross-linking via covalent bonding. This approach offers three benefits: (1) a means to optimize adhesive properties without the addition of an external photoinitiator; (2) the ability to switch off the tack adhesion on demand via a high cross-linking density; and (3) a platform for additional chemical modification. A series of poly(*n*-butyl acrylate-*co*-2,3,4,5,6-pentafluorobenzyl acrylate) or poly(PFBA-*co*-BA) copolymers were synthesized and modified post-polymerization into the photo-reactive poly(*n*-butyl acrylate-*co*-4-azido-2,3,5,6-tetrafluorobenzyl acrylate) [azide-modified poly(PFBA-*co*-BA)] with various molar contents. When cast into films, the azide-modified copolymers with a high azide content achieved a very high shear resistance after UV irradiation, whereas the tack and peel adhesion decreased strongly with the increase in azide content, indicating that excessive cross-linking occurred. These materials are thus photo-switchable. However, in the low range of azide content, an optimum probe tack adhesion energy was obtained in films with a 0.3 mol % azide content, where a long stress plateau (indicating good fibrillation) with a high plateau stress was observed. An optimum peel adhesion strength was achieved with 0.5 mol % azide. Thus, the adhesion was finely controlled by the degree of cross-linking of the PSA, as determined by the azide content of the copolymer chain. Finally, as a demonstration of the versatility and advantages of the material platform, we show an azide–aldehyde–amine multicomponent modification of the azide copolymer to make a dye-functionalized film that retains its adhesive properties. This first demonstration of using azide functionality has enormous potential for functional PSA design.

## Introduction

Pressure-sensitive adhesives (PSAs) are viscoelastic materials that can be adhered to a variety of substrates using light pressure.^1^ PSAs commonly find applications in tapes, labels, hygiene and medical products, and graphic films.^2^ They can be made of natural or synthetic rubbers, acrylics, silicones, polyurethanes, ethylene–vinyl acetate copolymers, polyethers, or polyesters.^3^ Among the compositions, acrylics are the most versatile and widely used polymers to produce PSAs.

PSAs require a balance of viscous and elastic properties for optimal adhesion. The elastic component of the dynamic shear modulus (*G*′) resists flow under shear stress and allows clean debonding from the substrate surface, but it must be low enough to allow conformal contact with the substrate. At the same time, the viscous component of the modulus (*G*″) must be low enough to allow adequate wetting of a substrate yet high enough to dissipate energy during the adhesive bonding.^4,5^ Dahlquist^6^ stated that the *G*′ must be less than 0.1 MPa at the bonding frequency (typically 1 Hz) to allow a PSA to wet a substrate and to form close contact. Deplace et al.^7^ proposed the maximization of the ratio of the loss tangent (tan δ = *G*″/*G*′) to *G*′ to achieve the greatest tack adhesion via fibrillation. Furthermore, Zosel^8^ proposed that fibrillation during debonding (dissipating energy) requires that the molecular weight between entanglements (*M*_e_) is more than 10–15 kg/mol, whereas polymers below this range debond with a sharp decrease in stress at a low strain, resulting in a decreased tack and adhesion energy. The *M*_e_, which is inversely related to *G*′, must not be too low to satisfy the Dahlquist criterion. A low chain entanglement density, which is achieved with a low *M*_w_/*M*_e_ ratio, reduces *G*′. Hence, higher *M*_e_ values yield higher viscoelastic energy dissipation during debonding and a greater viscous flow during bonding to increase the tack adhesion.^9^ For poly(acrylates), Tobing and Klein^9^ found a maximum adhesive peel strength when *M*_w_/*M*_e_ was approximately 10.

It is known that acrylic copolymers without chemical and/or physical cross-linking result in poor thermomechanical stability and are impractical as PSAs.^10^ Light cross-linking is used in PSAs to impart strain-hardening during fibril extension (leading to a clean detachment from the substrate) and to achieve a high resistance to shear stress.^7^ Cross-linking in acrylic PSAs can be accomplished through the addition of di-/trifunctional crosslinkers such as isocyanate crosslinkers (which form covalent bonds with hydroxy-functional monomers)^11^ or metal acetylacetonates (which can be chelated by acrylic acid comonomers).^12,13^ In contrast, photo-cross-linking is typically achieved through the incorporation of photo-initiators based on benzophenone, anthraquinone, or fluorenone^14,15^ for ultraviolet (UV)-cross-linkable PSAs. 4-Acryloyloxybenzophenone is a common example and is typically employed in a feed of 0.5 mol %.^16,17^ Upon UV irradiation, the benzophenone groups form radicals which abstract hydrogen atoms from nearby C–H bonds. The radicals can migrate through further hydrogen abstraction and the formation of more stable radicals, which result in cross-linking (through recombination of two radicals) at sites different than the benzophenone group.^18^ If no CH group is available nearby, the excited benzophenone group relaxes without cross-linking.^19^

Perfluorophenylazides, by comparison, irreversibly form a highly reactive nitrene upon UV irradiation, which can insert into nearby C–H, C=C, or N–H bonds or add onto N- or S-based lone pairs.^20^ While this chemistry finds application in photoaffinity labeling and surface functionalization,^21^ it has, to the best of our knowledge, not been applied to produce PSAs. Recently, poly(4-azido-2,3,5,6-tetrafluorobenzyl methacrylate) has been prepared through the direct polymerization of the azide-functional monomer^22^ or through highly efficient azide–*para*-fluoro post-polymerization modification of poly(2,3,4,5,6-pentafluorobenzyl methacrylate)^23^ and was used for photo-cross-linking of diblock copolymer nanoparticles.^22^ Li et al.^22^ have also demonstrated multicomponent reactions using piperidine and morpholine as secondary amines on poly(4-azido-2,3,5,6-tetrafluorobenzyl methacrylate).

In addition to UV-cross-linkable PSAs, which have been extensively studied to optimize the adhesive properties,^24−27^ photo-irradiation has also been investigated for “debond-on-demand” adhesives for applications in the removal of wound dressings,^28,29^ semiconductor chip fabrication,^30,31^ and the recycling of labeled bottles.^32^ The adhesive strength is reduced after photo-cross-linking irradiation when *G*′ exceeds the Dahlquist criterion. The adhesives cross-link upon exposure to light, and adhesive properties of peel adhesion and tack are greatly reduced depending on the amount and type of photo-initiators, the number of reactive sites, and the UV dose (irradiation time and lamp power). For example, the copolymerization of PSAs containing 2-ethylhexyl acrylate, ethyl acrylate, acrylic acid, and 2-hydroxyl-2-methyl-1-phenylpropane-1-one photo-initiator, followed by modification with glycidyl methacrylate (GMA) to provide photo-cross-linkable groups in the side chain was conducted by Ryu et al.^30^ Increasing the GMA amount and UV dose resulted in a decrease in tack and peel strength due to the increase in cross-linking density upon UV radiation. Recent developments in “debonding-on-demand” photo-switchable adhesives have been reviewed by Hohl and Weder^33^ as well as by Bandl et al.^34^

In this work, we report an expedient synthetic strategy for soluble pre-polymers that are photo-cross-linked under UV radiation without the addition of a photo-initiator. Specifically, the acrylic analogue, 2,3,4,5,6-pentafluorobenzyl acrylate,^35,36^ is copolymerized with *n*-butyl acrylate (chosen for its low glass transition temperature) and converted post-polymerization into the photo-reactive 4-azido-2,3,5,6-tetrafluorobenzyl analogue. A systematic variation of the 4-azido-2,3,5,6-tetrafluorobenzyl content revealed optimal PSA behavior at a molar azide content of 0.3 mol %. Conversely, a high (17 mol %) azide content was found to allow for switching off the tack and peel adhesion properties through post-fabrication irradiation. To demonstrate the versatility of the material platform for chemical modification and properties design, we also show the use of the reactive azide-functional precursor in an azide–aldehyde–amine multicomponent reaction^37^ to covalently bind 4-nitro-7-(1-piperazinyl)-2,1,3-benzoxadiazole (piperazinyl-NBD) as a model dye.

## Experimental Section

### Materials

2,3,4,5,6-Pentafluorobenzyl bromide (Fluorochem, 99%) and acrylic acid (Sigma-Aldrich, 99%) were used as received. Acetone (Sigma-Aldrich, ≥99%) was stored with molecular sieves (3 Å, Sigma-Aldrich) to make it anhydrous prior to use. Diethyl ether (≥99.8%), *n*-butyl acrylate (BA, ≥99%), and methanol (≥99.9%) were purchased from Sigma-Aldrich and used as received. Potassium carbonate (anhydrous, ≥99%), sodium bicarbonate (≥99.7%), anhydrous magnesium sulfate, acetonitrile (≥99.8%), chloroform (≥99.8%), and tetrahydrofuran (THF, chromatography GPC grade) were obtained from Fisher Scientific. Sodium azide (99%) and *N*,*N*-dimethylformamide (DMF, dried over molecular sieves, 99.8%) were purchased from Acros Organics and used as received. Chloroform-D (CDCl_3_, 99.8%, +0.05% v/v TMS) was obtained from Cambridge Isotope Laboratories, Inc. Azobisisobutyronitrile (AIBN, Sigma-Aldrich, 98%) was recrystallized from methanol and dried at room temperature prior to storage in a freezer. 4-Chloro-7-nitro-2,1,3-benzoxadiazole (NBD chloride, 98%) and piperazine (99%) were purchased from Sigma-Aldrich and Fluorochem, respectively. Two commercial polyacrylate copolymer PSAs (referred to hereafter as P1 and P2), which were used as a benchmark in this work, were provided by Synthomer plc.

### Characterization

^1^H and ^19^F NMR spectra were recorded on a 400 MHz Bruker spectrometer. Samples were dissolved in CDCl_3_ containing tetramethylsilane (TMS) as an internal standard. Both spectra were used to determine the conversion of the monomer into a polymer. Fourier transform-infrared spectroscopy (FT-IR) was recorded on a PerkinElmer spectrum two spectrometer equipped with attenuated total reflectance (ATR) accessories via scanning in the 4000–500 cm^–1^ range at a resolution of 4 cm^–1^. Size exclusion chromatography (SEC) was performed on a Viscotek GPCMax VE 2001 setup using THF as eluent with refractive index (RI) detection. The kit was equipped with three linear 7.5 × 300 mm PLgel mixed-D columns operating at 35 °C and a flow rate of 1.0 mL/min and calibrated with PMMA standards (known molecular weights in the range of 0.875–1677 kg/mol). Reported molecular weights are PMMA-equivalent values. Samples with a polymer concentration of about 2–4 mg/mL were dissolved overnight in THF and filtered through 0.2 μm regenerated cellulose syringe filters before injection. Differential scanning calorimetry (DSC) experiments were conducted using a heat–cool–heat cycle between −100 and 50 °C under a nitrogen atmosphere at heating/cooling rates of 10 °C/min using a commercial spectrometer (DSC Q1000, TA Instruments, New Castle, USA). Approximately 2–5 mg of samples was weighed and sealed in an aluminum hermetic pan.

### Monomer Synthesis

2,3,4,5,6-Pentafluorobenzyl acrylate (PFBA) was prepared according to a literature procedure.^35^^1^H NMR (400 MHz, CDCl_3_): δ/ppm 6.45 (1 H, *H*HC=CHR), 6.12 (1 H, HHC=C*H*R), 5.89 (1 H, H*H*C=CHR), 5.29 (2 H, OCH_2_). ^19^F NMR (376 MHz, CDCl_3_): δ/ppm −141.8 (2 F, ortho), −152.5 (1 F, para) and −161.6 (2 F, meta). FT-IR ν/cm^–1^: 2975 (w, C–H stretch), 1732 (m–s, C=O ester stretch), 1659 (w, C=C stretch), 1505 (s, C=C stretch), 1130 (s, C–O stretch) and 1053 (s, C–F stretch) (see Figure S1).

### General Procedure for Free Radical Solution Polymerization

#### Synthesis of Poly(*n*-butyl Acrylate) or PBA

A mixture of *n*-butyl acrylate (BA, 100 equiv, 39 mmol, 5.65 mL), AIBN (1 equiv, 0.4 mmol, 64.1 mg), and acetonitrile (total volume approximately 2-fold volume of BA) was mixed in a round-bottom flask equipped with a magnetic stir bar. The flask was sealed with a septum and the mixture was degassed for 30 min by purging with nitrogen. The reaction mixture was placed into a preheated oil bath at 70 °C for 16 h. The reaction was quenched by exposing to air and cooling to room temperature, followed by the determination of monomer conversion using ^1^H NMR spectroscopy. The polymer was precipitated twice into an excess (approximately 20–30-fold in volume) of methanol, and the product was collected by centrifugation followed by drying in a vacuum oven at 40 °C. ^1^H NMR (400 MHz, CDCl_3_): δ/ppm 4.03 (2 H, OCH_2_), 1.59 (2 H, COOCH_2_*CH_2_*CH_2_CH_3_), 1.38 (2 H, COOCH_2_CH_2_CH_2_CH_3_) and 0.95 (3 H, COOCH_2_CH_2_CH_2_C*H_3_*). FT-IR ν/cm^–1^: 2959, 2875 (w, C–H stretch), 1730 (s, C=O ester stretch) and 1452, 1379 (m–w, C–H bend) (see Figure S2).

#### Synthesis of Poly(*n*-butyl Acrylate-*co*-2,3,4,5,6-pentafluorobenzyl Acrylate) or Poly(PFBA-*co*-BA)

A similar procedure described above was performed for the copolymerization of PFBA (varying equiv) and BA (100 equiv). ^1^H and ^19^F NMR spectroscopies were used to determine monomer conversions. ^1^H NMR (400 MHz, CDCl_3_): δ/ppm 5.18 (2 H, OCH_2_), 4.03 (2 H, OCH_2_), 0.93 (3 H, CH_3_). ^19^F NMR (376 MHz, CDCl_3_): δ/ppm −141.8 (2 F, ortho), −153.0 (1 F, para) and −161.9 (2 F, meta). FT-IR ν/cm^–1^: 2959, 2875 (w, C–H stretch), 1730 (s, C=O ester stretch), 1508 (s, C=C stretch), 1159 (s, C–O stretch) and 1060 (m, C–F stretch) (see Figure S3).

#### Post-Polymerization Modification of Poly(*n*-butyl Acrylate-*co*-2,3,4,5,6-pentafluorobenzyl Acrylate) with Sodium Azide [Azide-Modified Poly(PFBA-*co*-BA)]

Poly(PFBA-*co*-BA) was dissolved in DMF to make a 25 w/w % solution. Then, sodium azide (1.5 equiv based on PFB groups) was added and the mixture was stirred for 2 h at 70 °C. After cooling at room temperature, complete reaction was confirmed by ^1^H and ^19^F NMR spectroscopic measurements. The reaction mixture was filtered and precipitated into an excess (approximately 20–30-fold in volume) of cold water. The product was isolated by centrifugation followed by freeze-drying, yielding poly(*n*-butyl acrylate-*co*-4-azido-2,3,5,6-tetrafluorobenzyl acrylate).^23^

### Synthesis of Piperazinyl–NBD

Piperazinyl–NBD was prepared from piperazine (1.35 g) and 4-chloro-7-nitro-1,2,3-benzoxadiazole (1.0 g) according to a literature procedure.^38^ 1.16 g (93%), ^1^H NMR (400 MHz, CDCl_3_): δ/ppm 8.43, 6.30 (2 H, ArH), 4.10 (4 H, CH_2_NAr), 3.13 (4 H, CH_2_NH) (see Figure S4).

### Azide–Phenylacetaldehyde–Amine Multicomponent Reaction

Piperazinyl–NBD (0.50 mg, 2 μmol, 0.4 equiv) and phenylacetaldehyde (0.24 mg, 2 μmol, 0.4 equiv) were dissolved in 2.5 mL of chloroform. Azide-modified poly(PFBA-*co*-BA) containing 0.5 mol % azide groups (AZ-0.5) (128.91 mg, 5 μmol of azide groups, 1 equiv) was added. The mixture was heated to 50 °C and stirred for 19 h. After cooling at room temperature, a sample was withdrawn, diluted with CDCl_3_, and analyzed by ^19^F NMR spectroscopy which indicated the conversion of 30% of azide groups (75% reaction efficiency based on added amine and aldehyde) into the desired amidine functionality. The reaction mixture was precipitated into methanol (25 mL), and the product was collected by centrifugation in the form of a dark yellow viscous liquid.

### Preparation of PSA Solutions and Films

The polymer [poly(PFBA-*co*-BA) or azide-modified poly(PFBA-*co*-BA)] was dissolved in acetone (0.2 g per mL) and left in a roller mixer overnight prior to film casting for characterization. For the probe tack test, the solution was cast on glass substrates (76 mm × 52 mm × 1.35 mm microscope slides, Fisher Scientific) using a 200 μm stainless steel cube applicator (Sheen Instruments, Cambridge, UK), whereas for peel adhesion and static shear tests, the copolymer solution was applied onto poly(ethylene terephthalate) (PET) sheets (50 μm thickness; 150 mm × 180 mm area) using a 90 μm wire bar applicator. The cast solutions were dried at 90 °C for 3 min in a convection oven (Heratherm Oven, Thermo Scientific), to form a film.

### Photo-Cross-Linking of Polymer Films

Photo-cross-linking was carried out in an UV cross-linker (UVP CL-1000, Cambridge, UK) equipped with five UV lamp tubes (302 or 365 nm wavelengths, power of 8 W). Polymer films were placed in the chamber and exposed to radiation for 10 min at a maximum energy density setting of 1 J/cm^2^.

### Adhesive Properties Measurement

**Figure 1:**
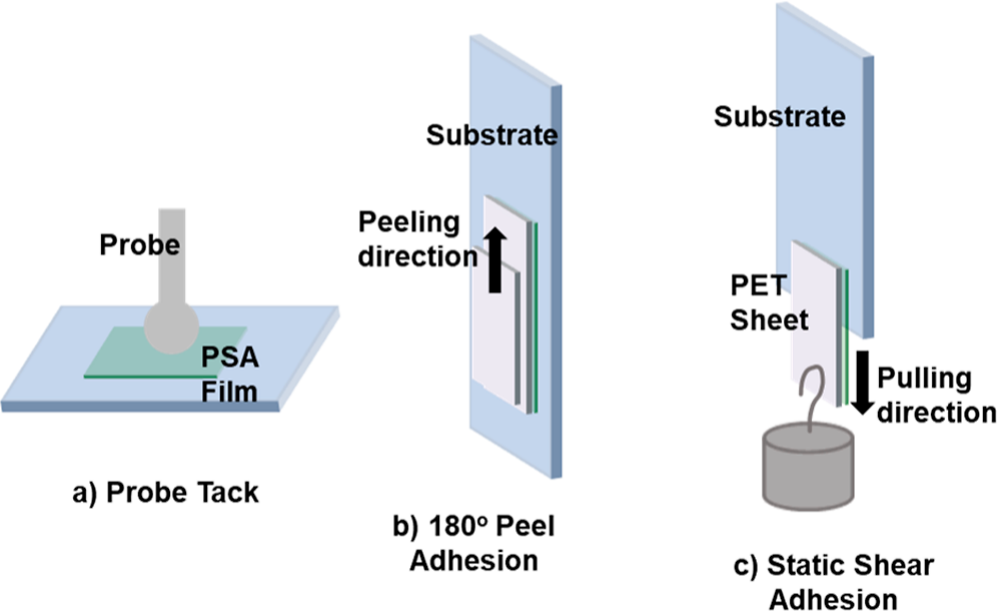
Illustration of experimental apparatus for adhesive properties measurements: (a) probe tack test; (b) 180° peel adhesion test; and (c) static shear adhesion.

The adhesive properties were determined for poly(PFBA-*co*-BA) and azide-modified poly(PFBA-*co*-BA) films, both before and after UV irradiation. Figure 1 illustrates the experimental tests of adhesive properties.

#### Probe Tack Testing

Polymer films were left to equilibrate for 10 min at 20.5 °C, 40% relative humidity (RH) prior to measurements. The probe tack measurement was conducted using both polypropylene and steel probes (diameter of 25.4 mm) on a mechanical testing apparatus (Texture Analyser, Stable Micro Systems, Godalming, UK). The surface of the probe was cleaned with acetone before each test. The probe was lowered onto the film at a velocity of 0.1 mm/s until a force of 4.9 N was reached, and then left in contact for a time of 1 s, before being withdrawn from the film surface at a constant velocity of 0.1 mm/s. For each type of probe, five measurements of each film were made both before and after UV radiation, and the mean values are reported here. The contact area (*A*) and thickness (*h*_0_) of the films were measured using a digital caliper. Dividing the force (*F*) by *A* to obtain stress (σ), and dividing the probe’s vertical displacement (Δ*l*) from the surface by *h*_0_ to obtain the strain, (ε), probe-tack curves were obtained. The tack adhesion energy was obtained from the area under the stress–strain curve as given by (eq 1)
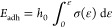
1

#### Peel Adhesion

180° peel tests were performed in accordance with the FINAT test method.^39^ Polymer films on PET sheets were allowed to equilibrate for 10 min at 20.5 °C, 40% RH. Then, a silicone release liner was applied to the free surface using a roller. Test pieces of the laminate were cut into 25 mm wide strips. After removing the release liner, the film was bonded to a steel plate, by rolling twice under the weight of a 2 kg roller. The sample was left for a 20 min dwell time. In some experiments, the peel test was performed immediately after; in other experiments, the films were irradiated through the PET sheet before the peel test. In this latter case, the sample was subjected to UV radiation at 365 nm for 10 min, and the peel test was performed immediately after. For each test piece, the peel adhesion at a 180° angle was measured by pulling the film back on itself at a velocity of 5 mm/s using a 5 kg load cell (texture analyser, Stable Micro Systems, Godalming, UK). The average of five measurements is reported here.

#### Static Shear Adhesion

Test pieces of the polymer film were cut into 25 mm × 175 mm areas, and the shear adhesion test was also performed in accordance with the FINAT test method.^39^ A 25 mm × 25 mm area of polymer film on PET sheet was bonded to a steel plate by rolling the film twice on to the surface under a weight of 2 kg roller. The sample was then exposed to UV irradiation through the PET film at 302 nm for 10 min. For each test piece, the steel plate was held at a 2° angle relative to a vertical plane, and a 1 kg weight was suspended on the free end of the test piece on a static shear tester (Model SS-RT-10, ChemInstruments, Ohio, USA). The time for each of the five test pieces to detach from the plate (i.e., the holding time) was recorded and averaged.

#### Gel Fraction Measurement

For gel fraction measurements, approximately 1.7 g of the copolymer solution (20 w/w %) was cast in a Petri dish and dried at 40 °C overnight in a vacuum oven to form a dry film of approx. 100 μm in thickness. Films were UV-irradiated in the same way as the films for tack characterization. Films were peeled from the Petri dish and placed into a cellulose extraction thimble. The gel fraction of these films (with an initial weight of *W*_1_) was determined by a Soxhlet extraction in boiling THF for 24 h. The insoluble polymer was dried at 40 °C for overnight in a vacuum oven and weighed (*W*_2_). The gel fraction, ϕ_gel_, was calculated as (eq 2)
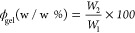
2

The procedure for determining molecular weight between crosslinks (*M*_c_) was slightly modified from that described by Tobing and Klein.^9^ After UV exposure, films were immersed in THF (1 w/v %) and shaken for 48 h, filtered on lens tissue (Whatman) to recover the gel, and the gel was dried in a vacuum oven at 40 °C for 3 h. The gel was then immersed in toluene and shaken for 22 h, filtered and the swollen gel was weighed while wet (*W*_3_), followed by drying in a vacuum oven at 40 °C for 24 h before the dry gel was weighed again (*W*_4_). The weight fraction of polymer (*W*_p_) swollen in toluene was calculated as
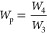
3

The volume fraction of polymer (ϕ) in solvent (toluene) was calculated as
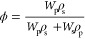
4where *W*_s_ is the solvent weight fraction, that is, 1 – *W*_p_, ρ_s_ is the density of solvent, toluene (0.8669 g cm^–3^), and ρ_p_ is the density of the dry polymer. Assuming ρ_p_ to be the density of PBA (1.06 g cm^–3^),^9^ the Flory–Rehner equation was used to calculate the average molecular weight between crosslinks, *M*_c_
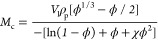
5where *V*_1_ is the molar volume of toluene (106.3 cm^3^ mol^–1^), and χ is the polymer–solvent interaction parameter, which was taken from the literature to be 0.34 (for PBA/toluene).^9^ The value of χ is subject to change when PBA is copolymerized with PFBA, but data are not available for the extent of the change in value. Consequently, swelling experiments provide only an estimate of *M*_c_.

## Results and Discussion

**Scheme 1:**
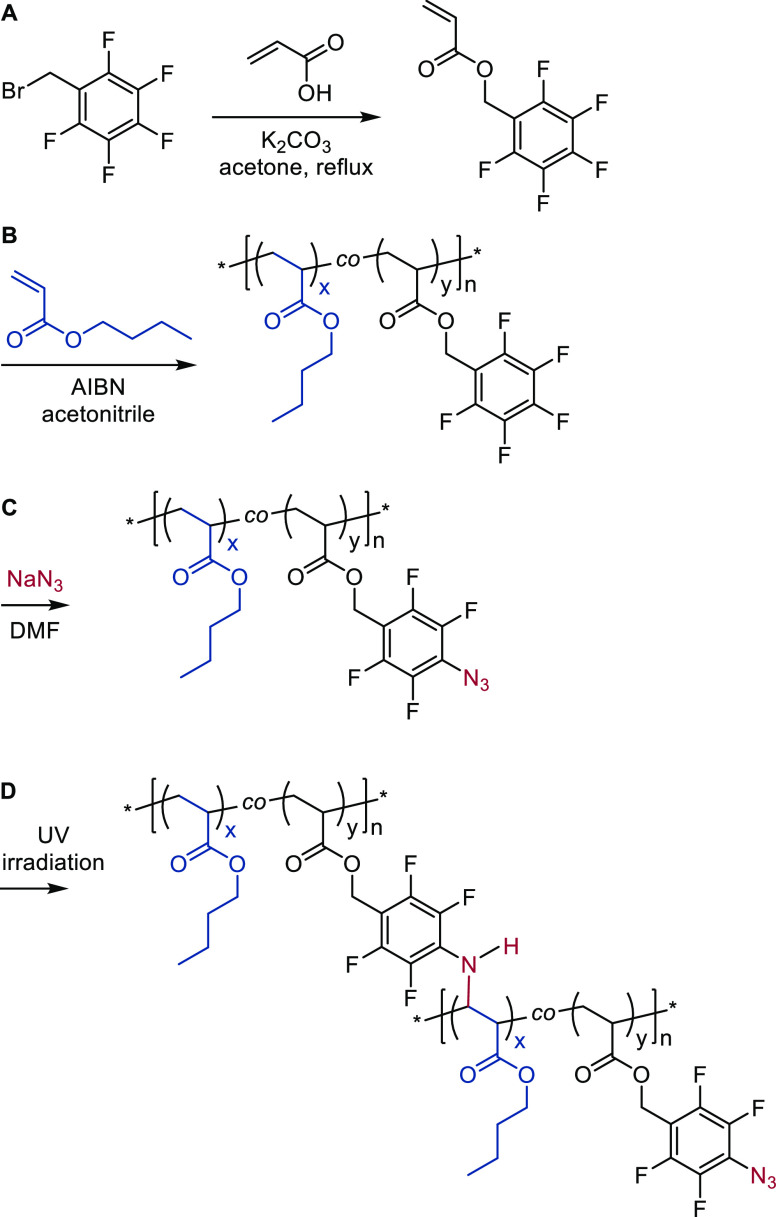
(A) Synthesis of the 2,3,4,5,6-Pentafluorobenzyl Acrylate (PFBA) Monomer, (B) Its Free-Radical Copolymerization with *n*-Butyl Acrylate (BA), (C) *para*-Fluoro Post-Polymerization Modification of Poly(*n*-butyl Acrylate-*co*-2,3,4,5,6-pentafluorobenzyl Acrylate) with Sodium Azide, and (D) Photo-Crosslinking (Nitrene Insertion Into a Backbone C–H Bond Shown Here)

2,3,4,5,6-Pentafluorobenzyl acrylate (PFBA) monomer was first synthesized (see Scheme 1A and Figure S1), followed by copolymerization with *n*-butyl acrylate (BA) via free-radical solution polymerization (Scheme 1B). Subsequently, the copolymers were modified post-polymerization with sodium azide to yield photo-reactive 4-azido-2,3,5,6-tetrafluorobenzyl analogues (Scheme 1C). Solutions of poly(*n*-butyl acrylate-*co*-4-azido-2,3,5,6-tetrafluorobenzyl acrylate)—denoted as azide-modified poly(PFBA-*co*-BA)—were cast into films and cross-linked by exposing them to UV radiation (Scheme 1D) to alter their adhesive properties. The properties of the azide-modified copolymers were compared to the unmodified poly(PFBA-*co*-BA) copolymers.

### Synthesis and Characterization of Poly(PFBA-*co*-BA) Copolymers and Azide Modification

A series of seven poly(PFBA-*co*-BA) copolymers with molar PFBA content ranging from 0.1 to 17 mol % (determined from the comonomer conversions measured by ^1^H and ^19^F NMR spectroscopy) were prepared in high isolated yields (>89%). See Scheme 1B. A PBA homopolymer was synthesized as a comparator (see Figure S2). Molar contents of all (co)polymers and their monomer conversions are summarized in Table 1, where the suffixes in codes represent the molar PFBA percentage. The syntheses were successful with a high monomer conversion achieved (>95%) in all cases. As an example of the synthesized produce, ^1^H NMR, ^19^F NMR, and FT-IR spectra of the copolymer with 17 mol % PFBA content (PFBA-17) are shown in Figure S3.

**Table 1:** List of Synthesized Copolymer Compositions

	feed		conversion^a^		PFBA content
code	BA (equiv)	PFBA (equiv)	BA (%)	PFBA (%)	PFBA (mol %)
high PFBA content					
PFBA-17	100	20	98.5	100	16.88
PFBA-9	100	10	99.0	>95	8.76
PFBA-5	100	5	98.6	100	4.83
PFBA-1	100	1	98.8	>95	0.95
low PFBA content					
PFBA-0.5	100	0.5	98.9	>95	0.48
PFBA-0.3	100	0.3	98.9	>95	0.29
PFBA-0.1	100	0.1	98.8	>95	0.09

a The percentage of conversion was calculated from ^1^H NMR for BA and ^19^F NMR for PFBA.

The molecular characteristics of the copolymers are presented in Table 2. For the purposes of discussing the adhesive properties in the sections that follow, the copolymers in the table are divided into two series: (1) high molar azide contents (1–17 mol %) and low molar azide contents (0.1–0.5 mol %). There is a general trend for the copolymers’ *M*_w_ to increase slightly with an increase in the PFBA content. The values, which are based on the measured PMMA-equivalent molar masses, range from 146 to 346 kg/mol.

**Table 2:** Molecular Weights (from SEC) and Glass Transition Temperatures of Poly(PFBA-*co*-BA) and Azide-Modified Poly(PFBA-*co*-BA)

code	*M*_n_ (kg/mol)	*M*_w_ (kg/mol)	*D̵*	*T*_g_ (°C)
PBA	80.1	156.5	2.0	–44
poly(*n*-butyl acrylate-*co*-2,3,4,5,6-pentafluorobenzyl acrylate) [poly(PFBA-*co*-BA)]				
PFBA-17	36.3	239.9	6.6	–29
PFBA-9	45.8	293.6	6.4	–36
PFBA-5	32.6	272.5	8.4	–39
PFBA-1	88.8	346.3	3.9	–43
PFBA-0.5	87.7	164.0	1.9	–44
PFBA-0.3	78.5	146.0	1.9	–45
PFBA-0.1	94.3	181.1	1.9	–46
poly(*n*-butyl acrylate-*co*-4-azido-2,3,5,6-pentafluorobenzyl acrylate) [azide-modified poly(PFBA-*co*-BA)]				
AZ-17	50.4	364.8	7.2	–27
AZ-9	64.2	371.7	5.8	–33
AZ-5	43.6	338.8	7.8	–39
AZ-1	101.3	391.0	3.9	–43
AZ-0.5	70.8	111.9	1.6	–47
AZ-0.3	74.9	152.8	2.0	–46
AZ-0.1	90.2	144.3	1.6	–45

The *M*_e_ for PBA has been reported to be 20.8 kg/mol.^9^ Assuming this same *M*_e_ for the copolymer series, the values of *M*_w_/*M*_e_ range from 7 to 17 for the range of PFBA compositions. The mean value of *M*_w_/*M*_e_ is 11, which is in the optimum range for PSAs. These high molecular weights provide a high entanglement density with an associated viscoelasticity, as is required for PSAs. However, the high molecular weights also increase the viscous component to resist the fibril extension.^9^

The measured molecular weight dispersity, *D̵*, generally increases as the PFBA content increases (Table 2), with values ranging between about 2 and 8, suggesting the occurrence of side reactions (e.g., chain transfer to PFBA monomers or repeat units), although such side reactions have not been observed during controlled radical polymerization of PFBA.^35^ The lower molecular weight fraction in a distribution is known to have the effect of reducing the entanglement network and thus reducing the *G*′ in the plateau region.^9^

It can also be seen in Table 2 that the *T*_g_ of (co)polymers increased from −46 °C (for PFBA-0.1) to −29 °C (for PFBA-17) as the PFBA content increased. Typically, a high-performing PSA has a *T*_g_ greater than approximately −40 °C.^40^ It is apparent that the PFBA content can be used to adjust the *T*_g_ value. In summary, the copolymerization of increasing amounts of PFBA has several effects that can be used to adjust PSA properties, including raising *M*_w_, *D̵*, and *T*_g_.

**Figure 2:**
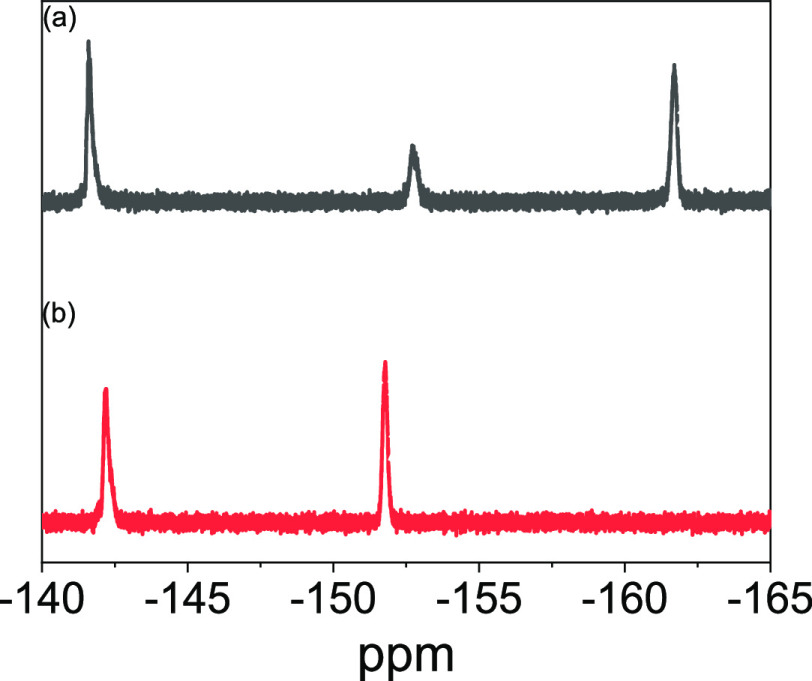
^19^F NMR spectra of (a) PFBA-9 copolymer and (b) after azide modification (AZ-9).

**Figure 3:**
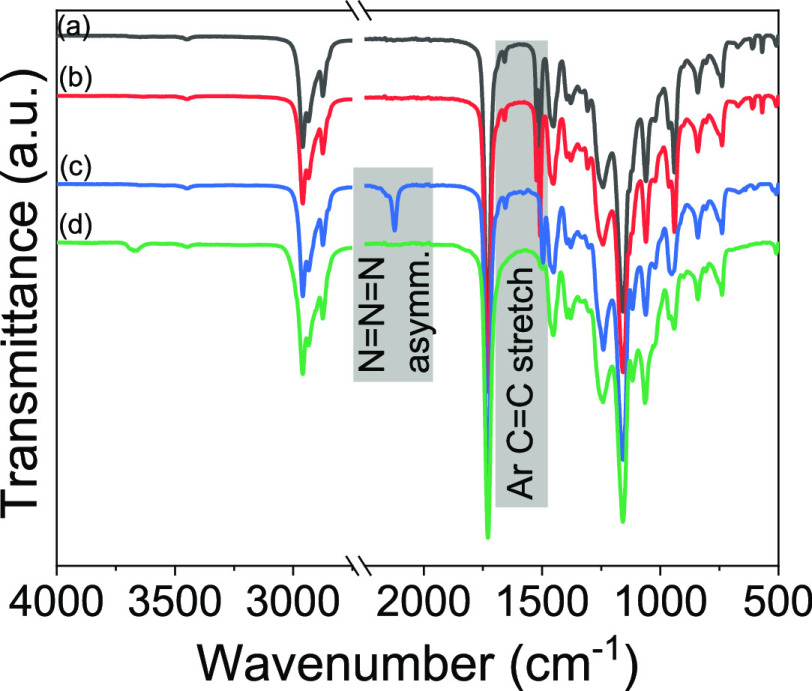
FT-IR spectra (normalized to the carbonyl peak and shifted vertically for clarity) for (a) PFBA-9 before UV radiation and (b) after UV radiation at a maximum wavelength of 302 nm for 10 min; (c) AZ-9 before UV radiation and (d) after UV radiation at 302 nm wavelength for 10 min.

Subsequently, a post-polymerization azide–*para*-fluoro substitution on poly(PFBA-*co*-BA) was performed,^23^ see Scheme 1C. Complete modification was confirmed through the disappearance of the *para*-F signal in the ^19^F NMR spectra and appearance of the signals [−142.21 ppm (2 F, ortho) and −151.82 ppm (2 F, meta)] associated with the desired 4-azido-2,3,5,6-tetrafluorobenzyl groups. See Figure 2. The successful reaction was further confirmed by the presence of a strong IR absorbance peak at 2124 cm^–1^ assigned to the asymmetric N=N=N stretching vibration of azide (Figure 3). The SEC-measured molecular weights, dispersities, and measured glass transition temperatures of the azide-modified copolymers are presented in Table 2, where an AZ prefix is used in the code names, and the suffix represents the azide content (mol %). In general, the broad trends in *M*_w_ and *D̵* for the copolymer series are retained in the corresponding azide-modified copolymers. For most samples, the SEC-measured *M*_w_ increased slightly with the azide–*para*-fluoro substitution, which reflected a change in the hydrodynamic diameter of the polymers in the SEC eluent (it should be noted that SEC separates by size and not by mass). For all copolymers, the *T*_g_ values for the azide-substituted polymers remained broadly unchanged compared to the copolymer with modest increases observed for AZ-9 and AZ-17.

### UV Cross-Linking of the Azide-Modified Poly(PFBA-*co*-BA)

Experiments were conducted to establish that the reaction of the azide functionality was successful in creating covalent cross-links in deposited films. FT-IR transmittance peaks of poly(PFBA-*co*-BA) (before azide modification) at all ranges of molar content (PFBA-1 through 17) before and after UV radiation were similar to each other, see Figure 3a,b. Moreover, the gel fraction of PFBA-17 was found to be <1 wt % after irradiation (Table 3), which indicated that no cross-linking reaction occurred in the absence of the azides. In contrast, FT-IR spectra for the azide-modified poly(PFBA-*co*-BA) showed the signatures expected for the azide reaction following UV radiation. Comparing Figure 3c,d, it is seen that the peaks for the azide functional group at 2124 cm^–1^ and the aromatic C=C stretching vibration at 1505 and 1659 cm^–1^ decrease, implying that the azide functionality was decomposed into nitrene, thus leading to intermolecular cross-linking (Scheme 1D). A gel fraction of 32 wt % was attained for the AZ-0.3 copolymer, and 75 wt % for the AZ-17 copolymer following irradiation. From swelling measurements after the UV exposure, estimates of *M*_c_ were obtained. *M*_c_ was 53 kg mol^–1^ for AZ-17 (derived from an experimental ϕ = 0.066), whereas the *M*_c_ for AZ-0.3 was higher with a value of 156 kg mol^–1^ (ϕ = 0.036), as is expected because of its lower azide content. Taken together, these data confirm that the azide cross-linking reaction successfully formed a covalently cross-linked network through nitrene insertion reactions upon UV irradiation.

**Table 3:** Gel Fraction of Copolymers before and after Exposure to UV Radiation (302 nm for 10 min)

	gel fraction, ϕ_gel_ (wt %)	
sample code	before UV radiation	after UV radiation
PFBA-0.3	<1	<1
AZ-0.3	<1	32
PFBA-17	<1	<1
AZ-17	9	75
P1^a^	23	
P2^a^	34	

a Commercial (non photo-reactive) samples included as benchmarks.

### Adhesive Properties of PSAs with a High Azide Content

Having synthesized the novel copolymers and established network formation via UV cross-linking, the adhesive properties (tack, peel, and shear strengths) were investigated.

Tack is evaluated during the debonding process. The formation and growth of fibrils during bond separation has been reported to cause high tack due to the adhesive’s ability to dissipate a large amount of energy during debonding.^8^ As an example of an optimized PSA, Figure S5 shows probe tack curves for the commercial PSAs (P1 and P2). Unsurprisingly, the probe-tack curves are virtually identical before and after UV exposure because the poly(acrylate) copolymer does not contain any reactive groups. To help with interpretation of the data, we recall the report of Creton and Lakrout^41^ on the deformation mechanism of the stress–strain curve using video observation across several stages of probe debonding. The first stage is the homogeneous deformation of the film, where the force increases rapidly with displacement and no cavities are optically visible. This is followed by the nucleation of cavities at the interface between the adhesive film and the probe and continues until the stress is no longer energetically able to withdraw the adhesive film, that is, the maximum tack stress (σ_max_) is reached. After reaching σ_max_, there is a sudden decrease in stress, in which simultaneous expansion of these cavities and interfacial cracks of cavities occur. Then, the stress levels at a nearly constant value, called the plateau stress (σ_plateau_), where the cavities grow in the lateral direction (in the plane of the film) and form a fibrillar structure. Finally, the fracture of the fibrils from the probe, defined as a strain at failure (ε_failure_), occurs by either debonding from the probe (adhesive failure) or by breaking (cohesive failure). The probe tack parameters for P1 and P2 are presented in Table 4 as a benchmark for comparison to our results.

**Table 4:** 180° Peel Adhesion and Probe Tack Properties of PBA and Commercial PSAs

sample code	peel adhesion (N/25 mm)	tack adhesion energy (J m^–2^)	maximum tack stress (MPa)
PBA	4.4 ± 0.3	48.1 ± 2.7	0.51 ± 0.03
P1	28.9 ± 0.6	107.9 ± 4.2	0.80 ± 0.02
P2	15.8 ± 0.6	103.6 ± 2.4	0.54 ± 0.01

Of the two copolymer series, the high PFBA content series was investigated first. The tack properties of the poly(PFBA-*co*-BA) copolymers with high (1–17 mol %) PFBA content before and after UV radiation showed a liquid-like mechanical response, demonstrated via a long downward sloping fibrillation region, gradually falling to zero via cohesive failure (Figure S6a,b). These results indicated that despite having a high molecular weight and being entangled, the polymers were too liquid-like to function as a PSA. Before irradiation, the non-crosslinked copolymers lacked cohesion. With an increase in the molar PFBA content, there was an increase in the initial slope of the probe tack curve (at low strains), suggesting an increase in the elastic modulus. This trend correlated with the hardening indicated by the rise in *T*_g_ with increased PFBA content. After UV radiation, the tack adhesion energies and maximum tack stress decreased slightly (Figure S6c,d). Decreasing in maximum tack stress after UV radiation indicated that the level of stress at which the cavity nucleated in the adhesive layer was affected by UV radiation.

**Figure 4:**
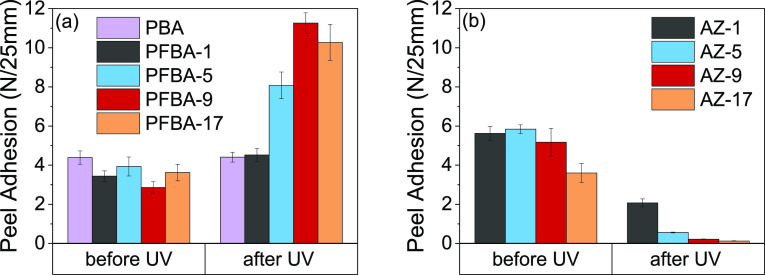
Peel adhesion strength of (a) PBA and poly(PFBA-*co*-BA), and (b) azide-modified poly(PFBA-*co*-BA) with high molar contents (as shown in the legend), before and after the UV irradiation of bonded specimens at 365 nm (10 min).

The copolymerization of BA with the increase in amounts of PFBA showed no effect on peel adhesion before exposure to UV radiation, with values remaining in the narrow range between 3 and 4 N/25 mm. However, after UV radiation, the peel adhesion increased, with a value of 11 N/25 mm being reached for PFBA-9, as is illustrated in Figure 4a. This was an unexpected result most likely due to the presence of PFBA functional groups because the peel adhesion of the PBA homopolymer remained constant at 4 N/25 mm after being exposed to UV radiation. One explanation is that the UV radiation could produce radicals in the PFBA groups, resulting in branch formation or chain scission and consequent changes to the molecular weight distributions. The existence of shorter chains (below *M*_e_) will swell the network of entangled chains and will decrease the elastic modulus and increase viscous dissipation, which is favorable to greater fibrillation and adhesion energy. The molecular weight distributions of PFBA-17 were compared before and after UV irradiation at 302 nm. There was no detectable difference to support the hypothesis, and the increase in the peel adhesion remains unexplained.

It is worth noting that the PSAs before and after UV radiation at these molar contents demonstrated cohesive failure. The peel adhesion was lower than for the two commercial PSAs (Table 4). P1 had a ∼63% higher peel adhesion than PFBA-9 and PFBA-17, whereas P2 only had a ∼31% greater peel adhesion. Both commercial PSAs likewise revealed cohesive failure when peeled from the steel substrate.

**Figure 5:**
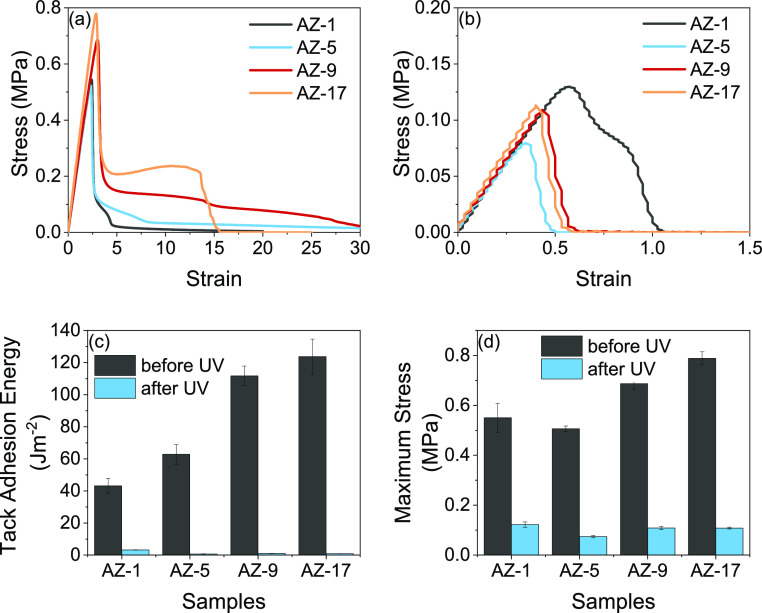
Representative probe-tack curves obtained using a polypropylene probe on azide-modified poly(PFBA-*co-*BA) films with high azide contents (as shown in the legend) (a) before UV radiation and (b) after UV radiation (notice the different range on the axes); (c) average tack adhesion energy and (d) maximum tack stress, comparing before and after UV radiation.

In contrast to these results for the high PFBA-content PFBA series, Figure 5a shows a positive effect of azide modification on the tack adhesion prior to the UV exposure. At the lower azide contents (AZ-1 and AZ-5), there was only weak fibrillation, but the fibrils extended to very high strain before failing cohesively. As the azide contents increased, the maximum tack stress and the stress plateau rose. The highest fibrillation plateau was observed for the AZ-17 copolymer. This copolymer showed a slight increase in the stress level of the plateau with the increase in strain, indicating there was strain-hardening before debonding of the fibrils from the substrate (adhesive failure). This probe tack curve bears similarities to the benchmark commercial adhesives (Figure S5). This strain-hardening is attributed to light cross-linking in the AZ-17 copolymer, which was confirmed by the presence of the gel (ϕ_gel_ = 9 wt %) in Table 3. The higher plateau value means that more energy was dissipated during the debonding,^42^ thus increasing the tack adhesion energy. Moreover, the stress carried by the fibrils is directly proportional to the elastic modulus of the material, with a stiffer adhesive having a higher plateau stress. This increase in the plateau stress correlates with an increase in *T*_g_. Incorporation of PFBA polar groups increased intermolecular forces and increases *T*_g_. The *T*_g_ values of copolymers after azide–*para*-fluoro substitution [azide-modified poly(PFBA-*co*-BA)] were slightly increased for AZ-9 and AZ-17 (Table 2).

When the azide-modified poly(PFBA-*co*-BA) polymers of the high azide content series were exposed to UV radiation, there was a pronounced loss of their adhesive properties, which is explained by the formation of heavily cross-linked networks and the resulting increase in shear modulus. In peel tests, the adhesives could be debonded with a light touch and there was failure at the interface (adhesive failure). The peel adhesion of bonded laminates dropped so low after UV irradiation that it could barely be measured, with values <1 N/25 mm for AZ-5, AZ-9, and AZ-17 (Figure 4b). This result suggested that the shear modulus, *G*, following the cross-linking exceeded the Dahlquist criterion of 100 kPa. With a measured *M*_c_ = 53 kg mol^–1^ for the AZ-17 copolymer after crosslinking, *G* was estimated to be 175 kPa according to the equation from rubber elasticity: *G* = ρ*RT*(1/*M*_e_ + 1/*M*_c_), where ρ is taken as the density of poly(butyl acrylate), 1060 kg m^–3^, *R* is the ideal gas constant, and *T* is taken as the room temperature. Thus, the loss of adhesion is consistent with the Dahlquist criterion.

The probe tack analysis similarly showed a loss of adhesion. After the UV cross-linking reaction and network formation, the adhesives no longer showed any fibrillation, and the probe-tack curves exhibited brittle debonding. Furthermore, after UV radiation, the maximum tack stress and strain at failure were much lower. Across the range of these higher azide content copolymers, both the tack adhesion energy and maximum tack stress dropped significantly after being exposed to UV radiation, as is shown in Figure 5c,d, respectively. As for the peel adhesion, their results are explained by excessive cross-linking that greatly increased *G*′ such that fibrillation was not possible.

Our results show that the adhesion of the azide-modified copolymers can be debonded on demand. The AZ-17 copolymer shows good tack and peel adhesion prior to UV radiation, but there is a nearly complete loss of adhesion following the UV radiation. This copolymer could find applications such as recycling when fast debonding on demand is needed.

Typically, PSAs with a high tack and peel adhesion strengths have low shear holding times and vice versa because the former requires extensibility, whereas the latter requires solid-like properties.^32,43^ The shear holding time of AZ-17, both before and after UV irradiation, was investigated. Results are tabulated in Table 5. Despite the fact that the tack and peel adhesion were significantly reduced after UV exposure, the shear holding time increased strongly. The adhesive did not fail after a time of 9753 min when the measurement was terminated. This result is explained by the loss of the dissipative component and an increased elastic modulus because of the cross-linked network formation, leading to solid-like behavior. Before the cross-linking, the viscous component of the copolymers is dominant so that there is flow under the shear stress. The shear holding time of AZ-17 was nearly three times higher than for the better-performing commercial PSA (P1). Thus, the perfluorophenylazide moiety is highly effective in making shear-resistant PSAs, although the tack and peel strengths are lost. In future research, a lower azide content could potentially be used to achieve greater tack and peel strength without sacrificing the high shear holding time. Peel and shear are typically opposing properties that require optimization in parallel.

**Table 5:** Static Shear Holding Time before and after Exposure to UV Radiation (302 nm for 10 min)

shear holding time (min)		
sample code	before UV radiation	after UV radiation
poly(PFBA-*co*-BA)		
PFBA-0.3	0.8 ± 0.1	0.9 ± 0.1
PFBA-17	3.5 ± 0.1	5.4 ± 0.6
azide-modified poly(PFBA-co-BA)		
AZ-0.3	2.8 ± 0.3	221.5 ± 3.5
AZ-17	6.7 ± 1.0	>9753.4
commercial PSA		
P1	3495 ± 269	
P2	530 ± 59	

### Adhesive Properties of PSA with a Low (0.1–0.5 mol %) Azide Content

As discussed above, with a high azide content, the tack and peel adhesion properties were switched off by UV radiation. Next, we investigated the effects of the less densely cross-linked networks in AZ-0.1, AZ-0.3, and AZ-0.5. Probe-tack experiments (using both steel and polypropylene probes) of this copolymer series exhibited liquid-like debonding with no stress plateau before and after UV irradiation, see Figures S7a and S8a. The tack adhesion energies obtained using the polypropylene probe (Figure S8c) were consistently lower than for the steel probe (Figure S7c) because the surface energy of the polypropylene probe (ca. 0.02 J/m^2^)^44^ was lower than the steel probe (ca. 0.5 J/m^2^).^45^ This indicated that high tack could be achieved if the substrate had a higher surface energy than the adhesive with good wetting.

**Figure 6:**
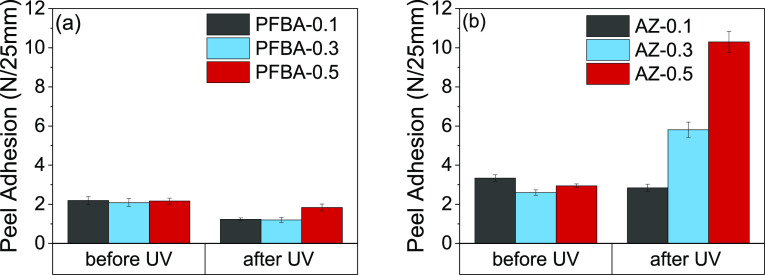
Peel adhesion strength of (a) poly(PFBA-*co*-BA) and (b) azide-modified poly(PFBA-*co*-BA) with low molar contents (as shown in the legend), comparing before and after UV radiation at 365 nm for 10 min.

The peel adhesion strengths of the poly(PFBA-*co*-BA) precursors and the azide-modified analogues with lower PFBA/azide content before UV radiation were found to be lower (2–3 N/25 mm) (Figure 6) compared to those with higher azide contents, as was shown previously in Figure 4 (values of 3–6 N/25 mm). The lower *M*_w_ copolymers with low azide content (AZ-0.1 through 0.5) should allow viscous flow, but these copolymers did not exhibit sufficient cohesion during the debonding process. In contrast, with the higher *M*_w_ in the higher PFBA and azide series (Figure 4), a higher viscosity and a more elastic response can explain the higher peel adhesion strengths.^9^

After UV irradiation, there was no significant change in the peel adhesion strength of the poly(PFBA-*co*-BA) series (shown in Figure 6a), which is expected for these copolymers without azides. However, the peel adhesion of the azide-modified poly(PFBA-*co*-BA) series rose after UV radiation by greater extents when the azide content was increased from 0.1 to 0.5 mol % (Figure 6b). The maximum peel adhesion strength (10 N/25 mm) was achieved with AZ-0.5. Cohesive failure was observed for this copolymer before and after UV exposure. These results show the effectiveness of the UV crosslinking. In comparison to the commercial PSAs, irradiated AZ-0.5 had a peel adhesion strength of about 35% of P1 and 63% of P2. Additional adjustments to the monomer composition and cross-linked network could be used to optimize the peel strength further.

**Figure 7:**
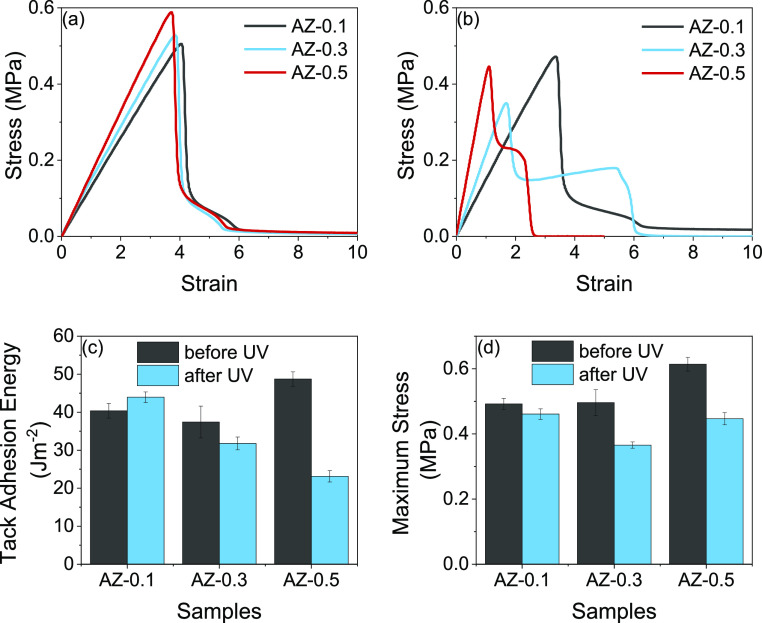
Representative probe-tack curves obtained using a polypropylene probe on azide-modified poly(PFBA-*co-*BA) films with low azide contents (as shown in the legend), (a) before UV radiation and (b) after UV radiation; (c) average tack adhesion energy; and (d) maximum tack stress, comparing before and after UV radiation.

Interestingly, after the azide-modified poly(PFBA-*co*-BA) films were exposed to UV radiation, the probe tack curves (Figure 7a,b) changed strongly, depending on the azide content. A long plateau region requires sufficient viscoelasticity to allow fibril extension. As the azide content increased, the plateau stress rose, while the fibrillation plateau shortened, indicating a loss of the viscous component. Notably, the AZ-0.3 copolymer showed a long plateau with some strain hardening during the fibril extension, followed by a clean detachment of the fibrils (i.e., adhesive failure). This constant stress is caused by a competition between the lateral growth of the cavities and the extension of the walls between the cavities in the direction leading to the fibrillar structure.^46^ The probe tack curve is comparable in shape to what is found for the benchmark PSAs (P1 and P2). In addition, there is evidence for a greater cross-linking density in the AZ-0.5 because the linear elastic region has a higher gradient, which is attributed to a higher elastic modulus. The effects of a higher modulus and reduced fibrillation are seen in the lower tack adhesion energy for AZ-0.5 after cross-linking (Figure 7c).

The shear holding time of AZ-0.3 was significantly increased by the UV cross-linking. Following radiation, the shear holding time was 221 min (Table 5), which is approximately one-half of the value for the commercial PSA, P2. Thus, the azide copolymers compare favorably with commercial adhesives and could be further optimized in future work.

### Azide–Phenylacetaldehyde–Amine Multicomponent Reaction on Azide-Modified Poly(PFBA-*co*-BA)

**Figure 8:**
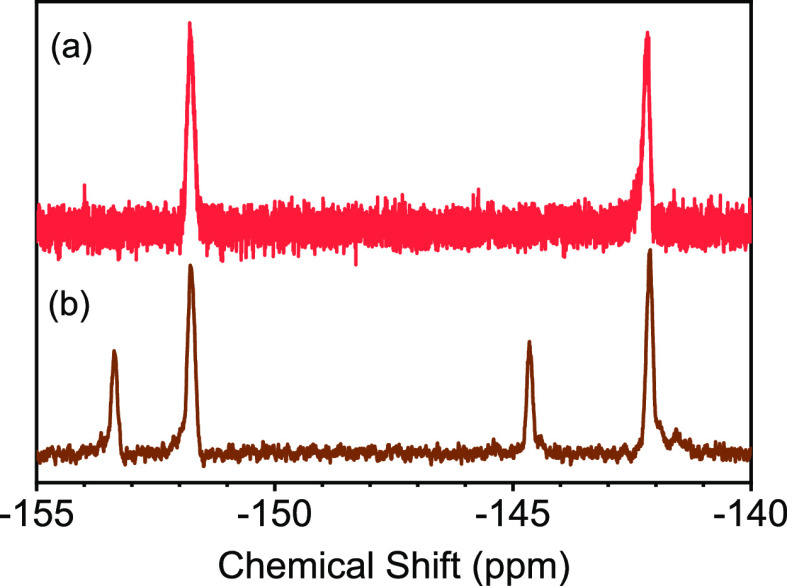
^19^F NMR spectra of (a) the reactive azide-functional precursor AZ-0.5 and (b) after partial multicomponent modification showing residual reactive azides and signals associated with the dye-functional aminidine groups.

**Scheme 2:**
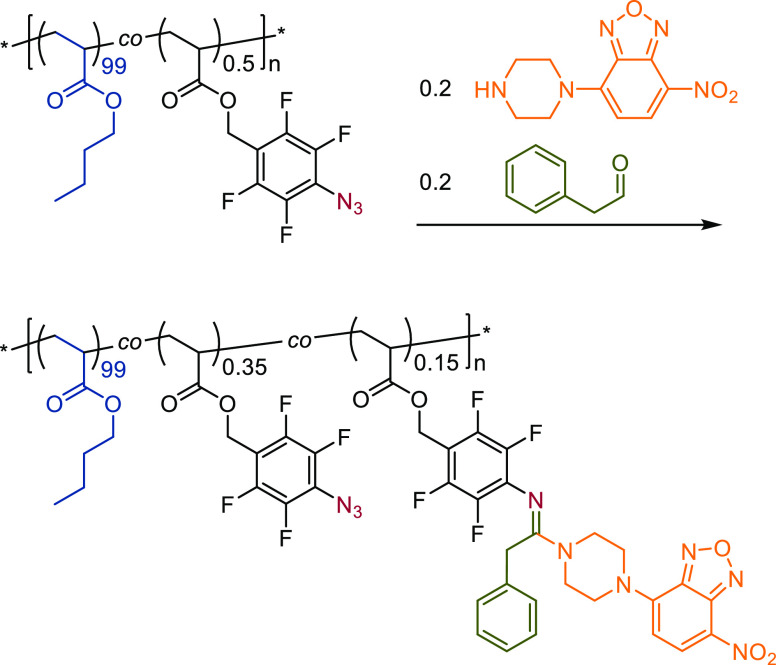
Partial Azide–Aldehyde–Amine Multicomponent Modification of Azide Groups on AZ-0.5 to Produce a Dye-Labeled Terpolymer Retaining UV Cross-Linkable Azide Functionality

After successfully using the perfluorophenylazide functionality for crosslinking and photo-switching of PSAs, we demonstrated that (unlike other photo-initiators used in PSA production) this reactive group also enables efficient post-polymerization functionalization. The azide–amine–aldehyde multicomponent reaction^37^ was chosen using phenyl acetaldehyde and 4-nitro-7-(1-piperazinyl)-2,1,3-benzoxadiazole (piperazinyl-NBD) as a model secondary amine carrying a yellow dye, see Scheme 2. Building on the optimal adhesive properties observed for AZ-0.3 (see above), AZ-0.5 was chosen for partial azide modification. A shortage (40% based on azide groups) of phenyl acetaldehyde and piperazinyl-NBD was reacted with AZ-0.5 to produce (at full conversion) a terpolymer retaining a total of 0.3 mol % of azide groups. Consistent with this strategy, after a reaction at 50 °C for 19 h, ^19^F NMR analysis showed signals at δ/ppm: = −144.7 and −153.4, characteristic of the desired amidine products.^22^ See Figure 8. Integration of the NMR signals indicated the conversion of approximately 30% of azide groups and a reaction efficiency of 75%, which is reasonably high given the low concentration of the three components. The terpolymer thus contained approximately 0.15 mol % of dye groups and 0.35 mol % of residual azide groups, close to the targeted azide content. The terpolymer was precipitated into methanol to remove unreacted dye and phenyl acetaldehyde to yield a brightly yellow colored material, visually confirming successful modification.

### Probe-Tack Analysis of Azide–Amidine Copolymer

**Figure 9:**
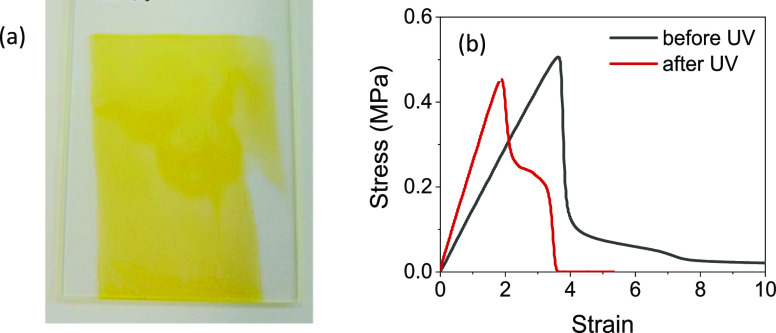
(a) Photograph of the dye-labeled azide–amidine terpolymer film showing a distinct color providing evidence for the modification. (b) Representative probe-tack curves obtained using a polypropylene probe on an azide–amidine terpolymer film, comparing before and after UV radiation.

The adhesive properties of the dye-labeled azide–amidine copolymer were studied. Films of the polymer appeared bright yellow (Figure 9a) due to the covalently attached NDB dye. Tack analysis of the azide–amidine polymer before UV radiation revealed a liquid-like mechanical response (Figure 9b), which was similar to what was reported for the AZ-0.3 earlier in this article. This indicated that the amidine modification did not affect the adhesion properties significantly. After UV irradiation, the probe-tack adhesion of the azide–amidine polymer was changed, as is shown in Figure 9b. The higher initial slope indicates a higher elastic modulus, and there is a plateau resulting from greater cohesion. There is a clean detachment at the end of the plateau, which is as expected for a lightly crosslinked polymer network. This result suggests that the residual azide in the dye-labeled terpolymer was still reactive and was able to be crosslinked. Increasing the amount of amidine groups could be used to deplete the reactive azides and thereby to tune the adhesion.

## Conclusions

We have reported here the first synthesis of poly(PFBA-*co*-BA) copolymers and their modification with azide functionality for use in a PSA. The azide—fluoro substitution took place at the *para*-fluoro position of PFBA as confirmed by NMR spectroscopy. Under UV radiation, the azide functionality successfully reacted to create a cross-linked network, which was confirmed by FT-IR spectroscopy, gel, and swelling measurements.

The azide content had a very strong effect on the adhesive properties—both before and after UV cross-linking. At an azide concentration of 17 mol %, the non-irradiated copolymer (AZ-17) showed acceptable tack and peel properties, which approached that of a benchmark. After UV cross-linking, the tack and peel adhesion were switched off to allow for easy removal. Thus, the azide copolymer can find applications as a “debonding on-demand” PSA. The solid-like copolymer, however, achieved an exceedingly high shear holding time. The tack and peel adhesion properties of the azide copolymers after UV cross-linking showed maximum values at very low azide concentrations of 0.3 and 0.5 mol %, respectively. At low azide content, the polymer is liquid-like, and the cross-link creates a gel that builds elasticity. At high azide content, the crosslinking raises the elastic modulus too high and adhesion is lost.

The azide-modified copolymer offers the distinct benefit of being a versatile platform for further chemical modification, such as through an azide–aldehyde–amine multicomponent reaction, as was demonstrated here using a dye as a model amine. In future work, we envisage using other chemistries available to the pentafluorobenzyl groups (such as reactions with thiols)^47^ and to the 4-azido-perfluorophenyl groups (which react very quickly with phosphines and with thioacids) to prepare PSAs having bespoke chemical functionality. These materials thus offer a strategy to modify a standard poly(acrylate) to optimize its adhesive properties via the azide content and to modify chemically with ease by the reaction of the azides.

## Supporting Information Available

^1^H NMR and IR spectra of PFBA; ^1^H NMR and FTIR spectra of PBA; ^1^H NMR and FTIR spectra of PFBA-17; ^1^H NMR spectrum of piperazinyl–NBD; probe-tack data of commercial adhesives (P1 and P2); probe-tack data of poly(PFBA-*co*-BA) films with poly(propylene) and steel probes; and molecular weights before and after UV irradiation (PDF)
