# Supplementary material for: Azide Photochemistry in Acrylic Copolymers for Ultraviolet Cross-Linkable Pressure-Sensitive Adhesives: Optimization, Debonding-on-Demand, and Chemical Modification
Source: ACS Appl Mater Interfaces. 2022 Jun 23;14(26):30216–27. doi: 10.1021/acsami.2c07385 (PMC9264322; doi:10.1021/acsami.2c07385)
Supplement: Supplementary file 1 — am2c07385_si_001.pdf [file am2c07385_si_001.pdf]

SUPPORTING INFORMATION

Azide Photochemistry in Acrylic Copolymers for Ultraviolet Cross-linkable Pressure-Sensitive Adhesives: Optimization, Debonding-on-Demand, and Chemical Modification

*Rohani Abu Bakar[1,2,3], Yuman Li[2], Oliver P. Hewitson[2], Peter J. Roth[2], Joseph L. Keddie[1]**

[1]Department of Physics, University of Surrey, Guildford, Surrey GU2 7XH, UK

[2]Department of Chemistry, University of Surrey, Guildford, Surrey GU2 7XH, UK

[3]Malaysian Rubber Board, Kuala Lumpur, 50450 Malaysia

*Corresponding author e-mail: j.keddie@surrey.ac.uk

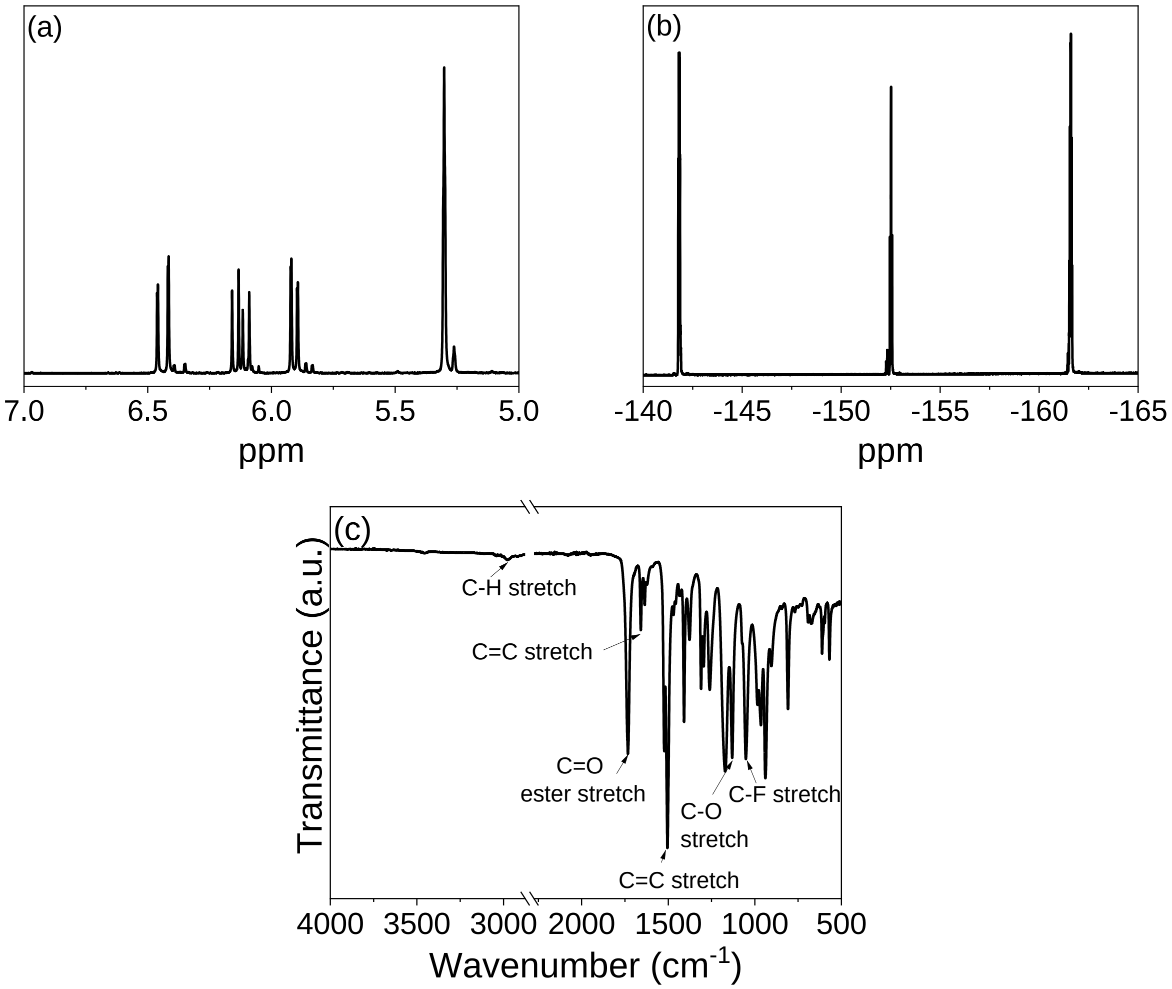

Figure S1. ^{1}H NMR and FT-IR spectra of 2,3,4,5,6-Pentafluorobenzyl acrylate or PFBA. (a) ^{1}H NMR (400 MHz, $CDCl_3$), δ/ppm: 6.45 (1 H, ***H***HC=CHR), 6.12 (1 H, HHC=C***H***R), 5.89 (1 H, H***H***C=CHR), 5.29 (2 H, OCH_2). (b) ^{19}F NMR (400 MHz, $CDCl_3$), δ/ppm: –141.8 (2 F, *ortho*), –152.5 (1 F, *para*) and –161.6 (2 F, *meta*). (c) FT-IR υ/cm^{-1} : 2975 (w, C–H stretch), 1732 (m–s, C=O ester stretch), 1659 (w, C=C stretch), 1505 (s, C=C stretch), 1130 (s, C–O stretch) and 1053 (s, C–F stretch).

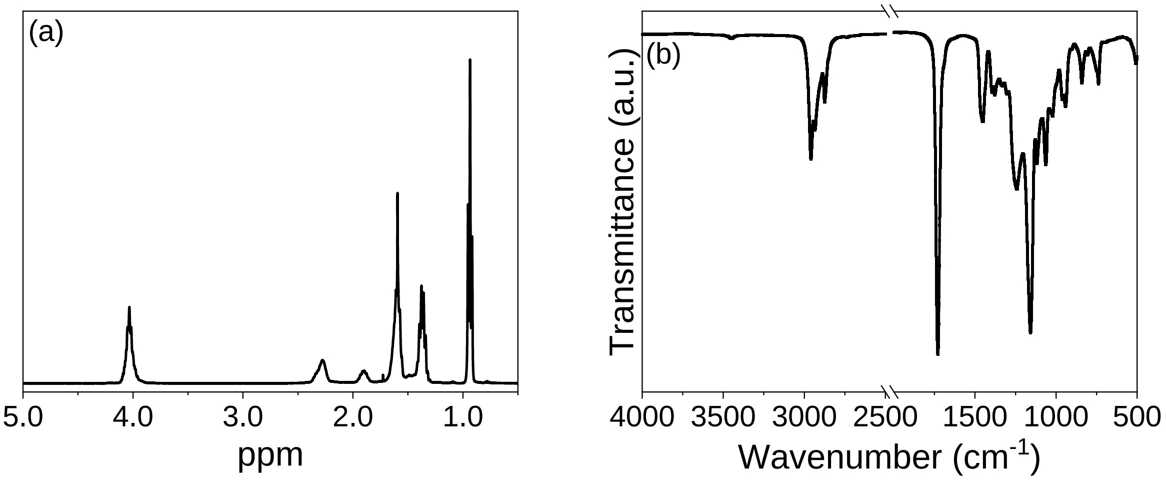

Figure S2. ^{1}H NMR and FT-IR spectra of poly(n-butyl acrylate) or PBA. (a) ^{1}H NMR (400 MHz, $CDCl_3$), δ/ppm: 4.03 (2 H, OCH_2), 1.59 (2 H, $COOCH_2C\boldsymbol{H_2}CH_2CH_3$), 1.38 (2 H, $COOCH_2CH_2C\boldsymbol{H_2}CH_3$) and 0.95 (3 H, $COOCH_2CH_2CH_2C\boldsymbol{H_3}$). (b) FT-IR υ/cm^{-1}: 2959, 2875 (w, C–H stretch), 1730 (s, C=O ester stretch) and 1452, 1379 (m–w, C–H bend).

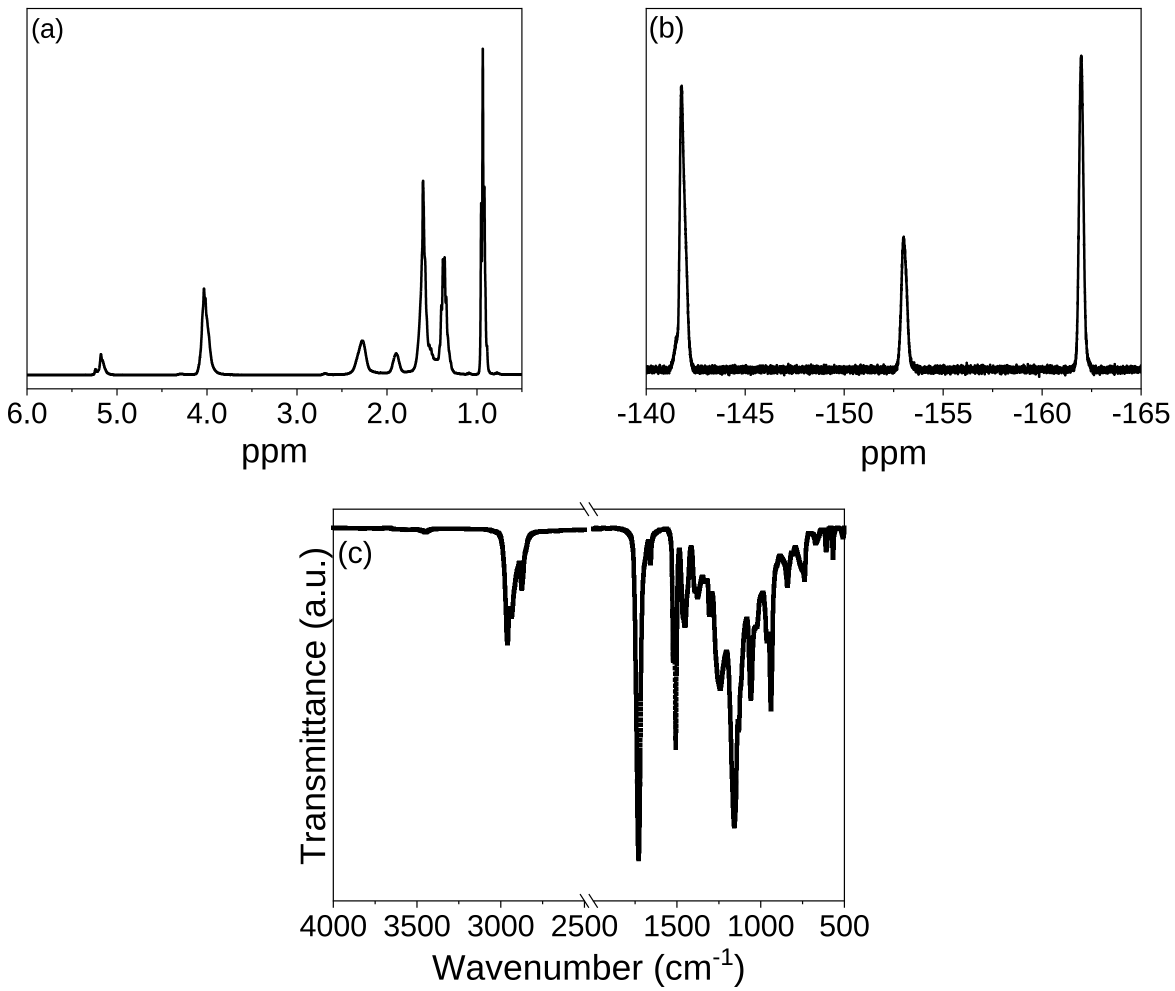

Figure S3. ^{1}H NMR and FT-IR spectra of poly(n-butyl acrylate-*co*-pentafluorobenzyl acrylate) with 17 mol-% PFBA (PFBA-17). (a) ^{1}H NMR (400 MHz, $CDCl_3$), δ/ppm: 5.18 (2 H, OCH_2), 4.03 (2 H, OCH_2), 0.93 (3 H, CH_3). (b) ^{19}F NMR (400 MHz, $CDCl_3$), δ/ppm: –141.8 (2 F, *ortho*), –153.0 (1 F, *para*) and –161.9 (2 F, *meta*). (c) FT-IR υ/cm^{-1}: 2959, 2875 (w, C–H stretch), 1730 (s, C=O ester stretch), 1508 (s, C=C stretch), 1159 (s, C–O stretch) and 1060 (m, C–F stretch).

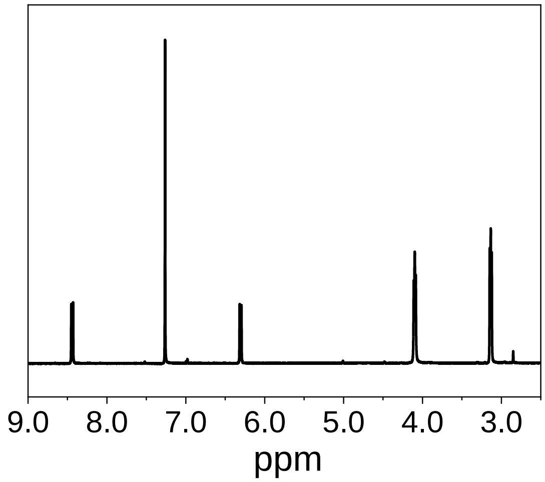

Figure S4. ^{1}H NMR spectrum of piperazinyl-NBD. ^{1}H NMR (400 MHz, $CDCl_3$), δ/ppm: 8.43, 6.30 (2 H, ArH), 4.10 (4 H, CH_2NAr), 3.13 (4 H, CH_2NH).

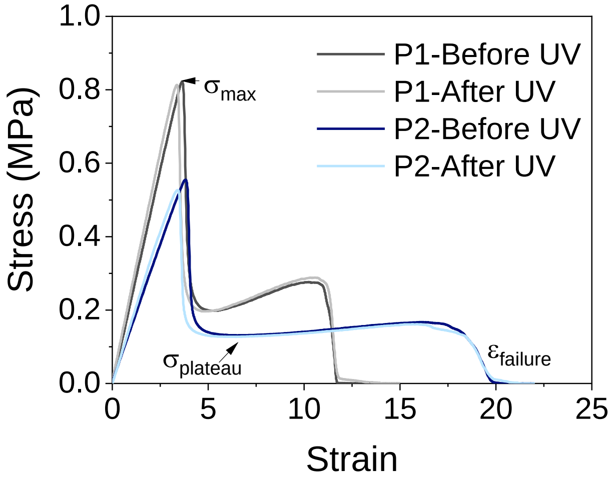

Figure S5. Probe tack curves of commercial P1 and P2 before and after UV radiation at 302 nm for 10 min.

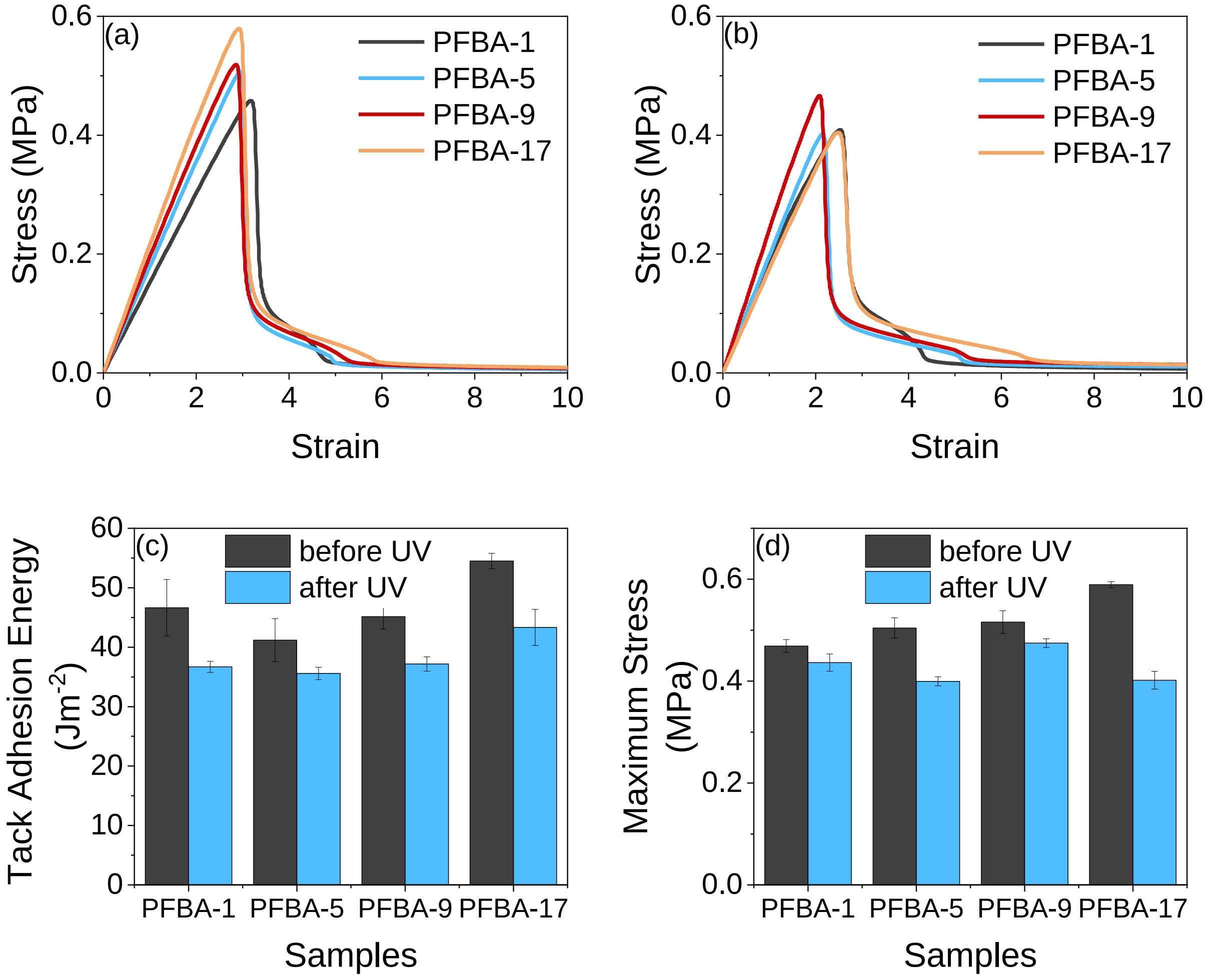

Figure S6. Probe tack curves obtained using a polypropylene probe on poly(PFBA-*co*-BA) films with high PFBA contents (as shown in the legend) (a) before UV radiation and (b) after UV radiation; (c) average tack adhesion energy and (d) maximum tack stress, comparing before and after UV radiation.

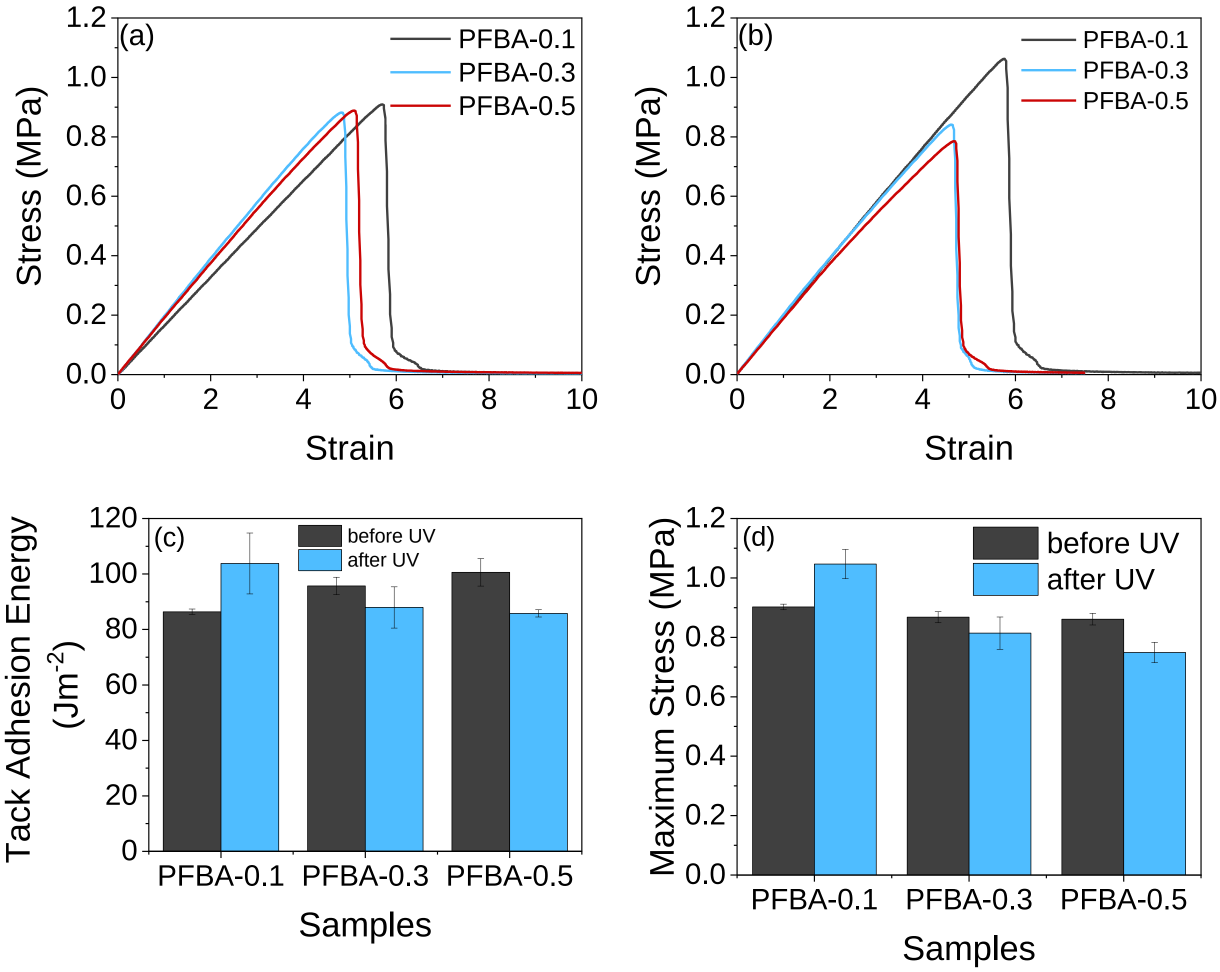

Figure S7. Probe tack curves obtained using a steel probe on poly(PFBA-*co*-BA) films with low PFBA contents (as shown in the legend) (a) before UV radiation and (b) after UV radiation; (c) average tack adhesion energy and (d) maximum tack stress, comparing before and after UV radiation.

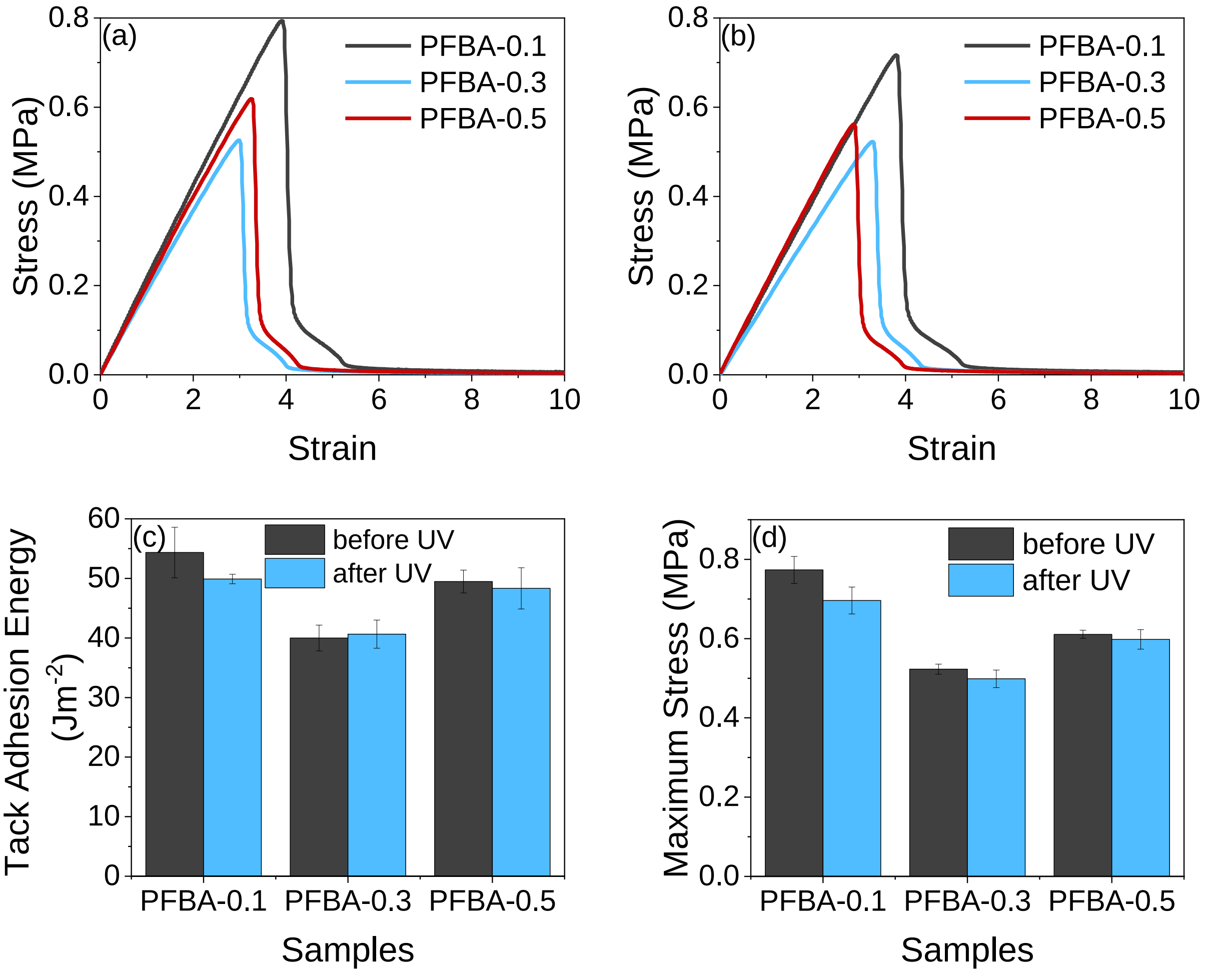

Figure S8. Probe tack curves obtained using a polypropylene probe on poly(PFBA-*co*-BA) films with low PFBA contents (as shown in the legend) (a) before UV radiation and (b) after UV radiation; (c) average tack adhesion energy and (d) maximum tack stress, comparing before and after UV radiation.
